# Transactions of the Chicago Gynecological Society, April 19th

**Published:** 1885-06

**Authors:** W. W. Jaggard

**Affiliations:** 2330 Indiana Ave.


### SOCIETY PROCEEDINGS.

---

TRANSACTIONS OF THE CHICAGO GYNECOLOGICAL SOCIETY.

*Stated Meeting, Friday Evening, 19th April, 1885.*

I.—FENGER. Chronic Peri-uterine Abscess, with the Demonstration of a Specimen.

II.—JAGGARD. Remarks upon a Teratom, with Exhibition of a Specimen.

The meeting was called to order by the President, Dr. H. P. MERRIMAN.

Professor CHRISTIAN FENGER narrated the following histories of three cases of chronic peri-uterine abscess:

CASE No. I. The patient, thirty-eight years old, married for a number of years, entered the Cook County Hospital one and one half years before the operation, with an attack of acute parametritis; pain and fever continued for five or six weeks, until the abscess burst into the rectum, and temporary relief was experienced. Pus, with periodic exacerbations of pain and fever, was discharged at intervals through the rectum until towards the end of the second year, when she entered the hospital for the second time.

*Status præsens.*—Temperature, morning, 101.2° F., evening, 103.5° F.; pulse, morning, 102, evening, 130. She is not emaciated, but there is œdema around the eye-lids.

On examination, a tumor, composed of plastic exudate, was found to the left and behind the uterus, pus was discharged with the fæces every two or three days; the communication between the rectum and abscess, surrounded by granulations, was found about three inches above the anal orifice. A tumor, extending three inches in every direction and reaching within one and one-half inches of the umbilicus, was found above the symphysis.

URINE; acid reaction, contains albumen and casts.

At no place in the vagina, could any prominence of the tumor be felt.

OPERATION; LAPAROTOMY. An incision in the median line revealed a red tumor, extending three inches in every direction. It was impossible to distinguish, by vision, between uterus and abscess. An aspirator needle was inserted into the supposed abscess, and two or three ounces of fœtid pus were removed. An incision was made with a knife through the uppermost portion of the abscess, the walls scraped, a drainage tube brought out through the vagina, and the sac united to the abdominal incision by two rows of sutures.

On the third day the temperature was 103° F., and blood oozed through the abdominal incision. The wound was opened and two or three ounces of bloody serum were removed from the right iliac fossa, and the drainage tube was shortened. Twelve days aftewards the temperature became subnormal and the woman died in wild delirium.

AUTOPSY; Peritoneal cavity healthy; drainage tube, one inch long, in the bottom of the sac, which did not communicate with the general peritoneal cavity. The spleen was slightly enlarged, and had undergone amyloid degeneration,—typical sago spleen. The kidneys normal in size, and of normal macroscopical appearance, showed evidence of amyloid change in the glomerules, upon application of the iodine test. When examined with the microscope, amyloid degeneration was perfectly plain. The cause of death was uræmia. The abscess was located in the uterine half of the left broad ligament, and the left ovary was missing. On the right side, the Fallopian tube was found embedded in a mass of connective tissue and the remains of an old hæmatocele. The Fallopian tube on the right side permits the entrance of a probe into the abscess cavity.

The operation was performed in the autumn of 1884.

CASE No. II. was one of acute parametritis, which occurred nine months prior to operation.

The abscess, situated high up behind the uterus, discharged into the rectum at periodic intervals; exacerbations of pain and fever were constant; progressive loss of weight and strength.

The tumor could be felt through the abdominal parietes, between the symphysis and umbilicus, presenting some of the appearances of the gravid uterus.

OPERATION; LAPAROTOMY. Upon incision through the median line, the same red tumor appeared. One ounce of pus was evacuated, and then it was noted that the interior of the sac was covered with a peculiar, cauliflower growth. The color was gray-white, not yellow. On this account, it was thought to be sarcoma.

This cauliflower growth was scraped out, and microscopical examination showed that it was tubercle (miliary) and not sarcoma. The cavity of the abscess was washed out, drainage effected through the vagina and the sac united to abdominal incision as in CASE No. I.

Within two weeks the tumor entirely disappeared, but a fistulous tract through the abdominal incision remained. This fistulous tract ultimately healed.

The tuberculous mass was entirely removed by scraping and subsequent washing out with potassium hydrate. One year later, the patient died. She had obstinate diarrhoea, and intestinal pain. The presumption is that she died from tuberculous ulceration of the intestine.

*Sections of the cauliflower growth, exhibiting giant cells and other morphological constituents peculiar to tubercle, were demonstrated to the Fellows.*

CASE No. III. was one of acute parametritis, which occurred four months before operation. The abscess opened into the rectum, as in the two former cases.

OPERATION; LAPAROTOMY. The abscess contained one pint of pus. The abscess cavity was treated as in the two former cases, with the exception that drainage was effected through the abdominal incision,

A serous cyst was also emptied. In six weeks, no trace of the abscess could be found, except a fistulous tract through the abdominal incision, which healed in three or four weeks.

REMARKS.—A chronic peri-uterine abscess may be tapped below the symphysis, through the vagina, or above the symphysis. Spontaneous cure, by opening into the bladder, rectum, or vagina, is always imperfect.

These abscesses, in the uterine half of the broad ligament, do not belong to the more common circumscribed collections of pus, pointing near Poupart's ligament.

When the abscess points through the vagina, the indication is clear.

When it does not, dissection through the vagina, along the posterior wall of the uterus, and forcing an opening into the cavity, is subject to the following objections:

I. Danger from hemorrhage. It is difficult to tell where large vessels in the abscess wall may be. The vessels are usually larger than normal. Then it is difficult to place a ligature around a bleeding vessel, as the uterus is fixed.

II. There is great danger of opening the general peritoneal cavity in dissecting up along the posterior uterine wall.

Laparotomy reveals the abscess in all its relations, and it is possible to prevent the access of pus to the abdominal cavity. The operation, devised by Lawson Tait, deserves election.

Schroeder has effected cures in two cases by the *sectio alta*, by dilating the communication between bladder and abscess. The communication between the abscess cavity and the viscus was old.

It is strange that few operators in Europe have practiced Tait's operation. Lawson Tait and a few Englishmen are the only surgeons, who advocate the measure.

Chronic peri-uterine abscesses of the character to which allusion has been made, are of frequent occurrence.

DISCUSSION.

Dr. EDWARD WARREN SAWYER, referred to a case of chronic peri-uterine abscess, seen in consultation with Dr. Charles Gilman Smith, in which enormous quantities of pus were discharged *per urethram*.

A vesico-vaginal fistula was made, and drainage through the *bas-fond* of the bladder into the abscess cavity was attempted The operation was abandoned, as it was found impossible to establish any communication by finger or catheter. In this case, there was an enormous dropsical swelling of the genitalia and leg on the right side, due, as suggested by Professor Fenger, to thrombosis of the iliac vein.

Professor E. C. DUDLEY inquired whether a quantitative examination of the urine for urea might not establish the fact of kidney disease, and consequently a contra-indication to operative procedure.

Professor FENGER replied that he did not know to what extent amyloid change might disappear. In cases of chronic suppuration, the liver may become enlarged, but it may become reduced in size again.

Albuminuria was only a relative contra-indication to operative procedure.

Professor C. E. DUDLEY related the histories of two cases of chronic peri-uterine abscess.

CASE No. I. was that of an abscess of the left broad ligament which opened into the vagina. The opening was dilated, and a double drainage tube inserted. The case was seen by Professer Fenger. The woman did well for a time, but after many ups and downs, died at the end of the year.

AUTOPSY. All the pus in the pelvic cavity was found well drained, but one small cavity, which contained about three ounces of horribly fetid pus, was found above the symphisis attached to the anterior abdominal wall. Lawson Tait's operation would, in all probability, have saved ber life.

CASE No. II. occurred in Mercy Hospital and presented many points in common with Professor Fenger's first case. Large quantities of pus were discharged *per rectum.* He urged Tait's operation.

The patient refused, and left the hospital in disgust, but returned within three months in blooming health, and showed no traces of the abscess. Tait's operation might have resulted fatally.

Professor W. W. JAGGARD raised the question as to the distinction between Tait's and Volkmann's operations. Dr. Franklin H. Martin had reported a case of pelvic abscess, treated by Volkmann's method, to the CHICAGO MEDICAL SOCIETY, during the past year. It did not differ essentially from the method pursued by Professor Fenger. He merely desired to suggest the subject of correct nomenclature.

PROFESSORS FENGER and DUDLEY thought that Dr. Martin's case was one of Tait's operation.

Professor W. H. BYFORD said that in all cases in which there is a tortuous communication between rectum, vagina, or bladder, there is an intermittent discharge of pus. Unilocular abscesses were the rule; multilocular abscesses, of the nature of Professor Dudley's CASE, No. I., were the exception.

Unilocular abscesses, as in Professor Dudley's CASE NO. II., have a tendency to spontaneous recovery. Professor Byford had demonstrated that these cases recover by granulation. The fungoid granulations spring up, and close in the abscess cavity. In other cases, the interior of the sac is glazed over with cicatricial tissue, serum fills up the cavity, and serous tumors are produced, which may disappear, but usually persist.

In extreme cases of chronic peri-uterine abscess, the question of operative procedure comes up.

Professor Byford was opposed to the line of treatment suggested by Professor Fenger. The *sphincter ani* should be dilated, or incised; the communication between abscess cavity and rectum dilated by fingers, steel dilator or knife, the cavity of the abscess scraped or washed out, and drainage effected *per rectum.*

Complete evacuation of pus, and sac-contents, could be effected in this manner without invasion of the general peritoneal cavity.

When the communication through the bladder was also established, he would treat the cases in the same way, or dilate the urethra, and proceed in the manner indicated.

After simple evacuation of purulent contents, abscess cavities do not always collapse. They may remain patulous. Their walls are frequently of great thickness and cartilaginous hardness.

It is usually necessary to scrape the cavity and establish a new action in the pyogenic membrane.

Schroeder's operation of dissecting up from below, through the vagina, along the posterior uterine wall, was justly considered dangerous.

Professor Byford had, on two or three occasions passed a sound into the rectum, cut down on the instrument, and kept the tract patulous by tents.

Professor CHRISTIAN FENGER closed the discussion by saying that he thought Professor Byford's suggestion as to drainage through the rectum impracticable when the opening was high up. It was always dangerous on account of the proximity of the general peritoneal cavity.

Professor E. C. DUDLEY asked "Does the tube remain *in situ* when the bowels are moved ?"

Professor BYFORD replied, "Yes."

II. Professor W. W. JAGGARD made the following remarks upon a teratom, with presentation of the specimen.

The teratom, or *portentum*, to which he desired to call attention, was sent for examination by Dr. J. L. Brenton and Dr. A. C. Helm, of Beloit, Wis.

He had not received any history of the case, and therefore merely called attention to the macroscopical characters of the object, reserving for a future occasion the discussion of the results of dissection.

The head presents a typical example of hemicephalus, or anencephalus, referable to Rokitansky's class A, deformities resulting from insufficient development.

Such fœtuses are often notable on account of remarkable development; the quantity of *liquor amnii* is usually large. They may assume longtitudinal or transverse presentations. When the vertex presents, the diagnosis is frequently of a very puzzling character.

CAZEAUX suggests as a valuable sign, under such circumstances, the convulsive movements of the child in the uterus, as the result of the direct irritation of the basal ganglia.

By this means, CAZEAUX and DUBOIS have been enabled to make a diagnosis before rupture of the membranes.

Version is usually indicated in these cases, if the presentation is that of vertex, not engaged.

The second object of interest, in connection with the teratom, is the large tumor of the ano-perineal region. Rokitansky refers these cases to class B, teratoms, as the result of pathological excess of development.

Tumors of the ano-perineal region are divided by Braune into the following classes:

(1) Tumors, after the exclusion of fœtation by inclusion, the connection of which with the special canal has been demonstrated.

(2) Degeneration of the gland of Luschka.

(3) Sacral Hygroma.

(4) Coccygeal tumors, fibromata, cysto-fibromata, cysto-carcinomata.

(5) Hydrorachis.

(6) Lipomata.

(7) Tumors, observed in adults, the congenital nature of which is not absolutely proven.

W. W. JAGGARD, M. D., Editor.

2330 Indiana Ave., 21st April, 1885.

